# Characterization and Functionality of Cellulose from Pomelo Fruitlets by Different Extraction Methods

**DOI:** 10.3390/polym14030518

**Published:** 2022-01-27

**Authors:** Chuanbo He, Hao Li, Jinling Hong, Hejian Xiong, Hui Ni, Mingjing Zheng

**Affiliations:** 1 College of Ocean Food and Biological Engineering, Jimei University, Xiamen 361021, China; hcbcc@jmu.edu.cn (C.H.); 13834702852@163.com (H.L.); 202012951063@jmu.edu.cn (J.H.); hjxiong@jmu.edu.cn (H.X.); nihui@jmu.edu.cn (H.N.); 2 Fujian Provincial Key Laboratory of Food Microbiology and Enzyme Engineering, Xiamen 361021, China; 3 Collaborative Innovation Center of Seafood Deep Processing, Dalian Polytechnic University, Dalian 116039, China; 4 Research Center of Food Biotechnology of Xiamen City, Xiamen 361021, China

**Keywords:** cellulose, pomelo fruitlet, alkaline hydrogen peroxide hydrolysis, acid hydrolysis, ultrasonication

## Abstract

Pomelo fruitlets have the potential for extracting cellulose. This study aimed to investigate characterization and functionality of cellulose extracted from pomelo fruitlets by different extraction methods. Cellulose extracted by acidic-alkaline hydrogen peroxide hydrolysis (CAA), alkaline hydrogen peroxide hydrolysis (CA), and ultrasonic assisted alkaline hydrogen peroxide hydrolysis (CUA) were prepared from pomelo fruitlets. The results showed that cellulose CUA had higher yield and purity with higher crystallinity and smaller particle size than those of CAA or CA (*p* < 0.05). Specifically, the yield of CUA was 82.75% higher than that of CAA, and purity was increased by 26.42%. Additionally, water- and oil-holding capacities of CUA were superior to those of CAA or CA, increasing by 13–23% and 10–18%, respectively. The improvement of water- and oil-holding capacities were highly related to its smaller particle size with increased surface area. The results suggested that ultrasonic assisted alkaline hydrogen peroxide hydrolysis is a promising and efficient method to prepare high-purity cellulose from pomelo fruitlets, and this cellulose is expected to be a food stabilizer and pharmaceutical additive.

## 1. Introduction

Cellulose is the world’s most abundant biopolymer with repeating D-glucose units linked by β-1,4 glycosidic bonds [1]. With various characteristics of non-toxicity, mechanical strength, biodegradability, and hydrophilic and hygroscopic nature, cellulose has been widely applied in many fields, including paper, textiles, pharmaceuticals, and food industries. [1,2]. In the food industry, cellulose is generally applied as food filler, thickener, or stabilizer for its emulsifying, foaming, film-forming, and encapsulation capabilities [1,3,4]. It is also explored as a functional ingredient because of its health benefits, such as hypoglycemic and hypolipidemic activity and bioavailability in intestine [1]. 

Numerous methods (such as alkaline hydrogen peroxide hydrolysis, acid hydrolysis, and hydrolysis assisted with ultrasonication) have been used to attempt separating cellulose from materials. Alkaline hydrogen peroxide hydrolysis is one of the most common approaches to extract cellulose [5,6]. Acid hydrolysis (e.g., using H_2_SO_4_ or HCl) is regarded as a safer method to obtain cellulose with fewer harmful effects on the environment [7,8,9]. Haafiz et al. (2013) [7] found that cellulose with 87% of crystallinity was extracted from oil palm biomass residue using 2.5 N HCl. In addition, cellulose nanofibers with long entangled network fibrils were successfully extracted from sugarcane bagasse through the combination of alkaline treatment and mild acid hydrolysis assisted with ultrasonication [10]. Nevertheless, the comprehensive study of cellulose prepared by different extraction methods on either the molecular or macroscopic level is seldom reported. In addition to extraction methods, different origins (e.g., cotton, petroleum, agri-food industry waste) also play a role in cellulose properties, including crystallinity, surface area, porous structure, and particle size [7,11].

Pomelo, a specialty citrus fruit, is abundantly harvested in Fujian Province, China [12,13]. Pomelo fruitlets are mainly generated from the pomelo growth process, the state of hormones in the reproductive organs, and removal of old or young leaves. They are normally discarded, resulting in 200,000~350,000 tons of waste every year. Studies have reported that pomelo fruitlets can be used to extract dietary fiber, polysaccharides, or flavonoids [14,15]. For example, Liu et al. (2021) [15] reported that dietary fiber prepared from pomelo fruitlet shows good anti-oxidative stress, hypoglycemic, and lipid-lowering abilities with observation of inhibiting glucose diffusion and α-amylase activity, decreasing cholesterol content, and increasing the abundance of *Firmicutes*, *Lactobacillus*, and *Prevotellaceae* in hyperglycemic mice. However, little attention has been paid to the cellulose of the pomelo fruitlet. Previous studies found that cellulose and cellulose nanocrystals can be prepared from pomelo peel to be explored as functional fibers for food applications [16,17]. Alkaline hydrogen peroxide hydrolysis was used to isolate cellulose from pomelo peel and to prepare microcrystalline cellulose, of which the water/oil binding capacity was much higher than that of commercial cellulose [16]. These findings suggest the potential for extracting cellulose from pomelo fruitlet to be a food stabilizer and pharmaceutical additive.

Therefore, cellulose samples were prepared from pomelo fruitlets by using different extraction methods, including acidic hydrolysis combined with alkaline hydrogen peroxide hydrolysis, alkaline hydrogen peroxide hydrolysis, and ultrasonic assisted alkaline hydrogen peroxide hydrolysis. Yields and purities of these prepared cellulose samples were compared, and their structural characterization and functionality (water- and oil-holding capacities, antioxidant activities in vitro) were investigated to define their applicability and suitability to develop new fields of research. The result can provide a scientific basis for the influence of different extraction methods on the molecular level or macroscopic properties of cellulose, further explore an efficient method for producing cellulose from pomelo fruitlets, and improve the high-value utilization of pomelo fruitlets.

## 2. Materials and Methods

### 2.1. Materials

Pomelo fruitlets were obtained from Zhangzhou Pomelo Township Food Co., Ltd. (Zhangzhou, China). Monosodium phosphate, sodium lauryl sulfate, ethylenediaminetetraacetic acid, concentrated hydrochloric acid (HCl), concentrated sulfuric acid (H_2_SO_4_), sodium hydroxide (NaOH), hydrogen peroxide (H_2_O_2_), and anhydrous magnesium sulfate were of analytical grade, and bought from Sinopharm Chemical Reagent Co. Ltd. (Shanghai, China).

### 2.2. Preparation of Cellulose from Pomelo Fruitlets

#### 2.2.1. Acidic-Alkaline Hydrogen Peroxide Hydrolysis

Cellulose was prepared from pomelo fruitlets through acidic-alkaline hydrogen peroxide hydrolysis, modifying from a previous study [18]. Pomelo fruitlets were washed and cut into 2 cm × 2 cm × 1 cm lumps, dried at 65 °C for 2 h, crushed into powder, and then passed through an 80-mesh sieve to obtain pomelo fruitlet powder. Pomelo fruitlet powder was added to 0.10 mol/L HCl solution at a ratio of 1:25 and heated for 150 min at 85 °C. Subsequently, the powder was filtered, washed, lyophilized, and crushed. To remove hemicellulose, the powder was treated with 10% NaOH solution at a ratio of 1:25 and heated for 80 min at 85 °C. The solution was filtered, washed, lyophilized, and crushed. Filtered residue was then mixed with 1.2% H_2_O_2_ solution at a ratio of 1:25 for 30 min at 30 °C. Cellulose prepared by acidic-alkaline hydrogen peroxide hydrolysis (CAA) was obtained after filtration, washing, lyophilization, and crushing.

#### 2.2.2. Alkaline Hydrogen Peroxide Hydrolysis

Cellulose was extracted from pomelo fruitlets using alkaline hydrogen peroxide hydrolysis according to a previous method with some modification [6]. Pomelo fruitlet powder was added into a mixture of 9% NaOH and 1% H_2_O_2_ at a ratio of 1:25 and heated for 240 min at 80 °C. Cellulose prepared by alkaline hydrogen peroxide hydrolysis (CA) was obtained after filtration, washing, lyophilization, and crushing.

#### 2.2.3. Ultrasonic Assisted Alkaline Hydrogen Peroxide Hydrolysis

Cellulose from pomelo fruitlets was extracted using ultrasonic assisted alkaline hydrogen peroxide hydrolysis as suggested by a previous study with some modification [19]. Pomelo fruitlet powder (2 g), ethylenediaminetetraacetic acid (0.02 g), and anhydrous magnesium sulfate (0.01 g) were added to a mixture of 1% H_2_O_2_ and 5% NaOH at the ratio of 1:25 and extracted for 40 min under 100 W ultrasonic power at 80 °C. Cellulose prepared by ultrasonic assisted alkaline hydrogen peroxide hydrolysis (CUA) was obtained after filtration, washing, lyophilization, and crushing.

### 2.3. Determination of Cellulose Yield and Purity

The yield of cellulose from pomelo fruitlets was calculated according to Equation (1),

(1) $$\mathrm{Yield}\;(\%)=\frac{w_{1}}{W}\times 100\%$$

where *w*_1_ is the weight of cellulose, g; and *W* is the weight of pomelo fruitlets, g.

Anthrone colorimetry was used to determine the weight of cellulose in product, and then the purity was calculated according to Equation (2),

(2) $$\mathrm{Purity}\;(\%)=\frac{w_{2}}{w_{1}}\times 100\%,$$

where *w*_1_ is the weight of cellulose product, g; and *w*_2_ is the weight of cellulose in cellulose product, g.

### 2.4. FT-IR Measurement

The FT-IR spectrum of cellulose from pomelo fruitlets was determined using a method reported previously [20]. Cellulose extracted from pomelo fruitlets (2 mg) was mixed well with potassium bromide (2 g, chromatographic purity), ground, and then pressed by vacuum compression. The samples were scanned in the range of 400–4000 cm^–1^ with a 4 cm^–1^ resolution via FT-IR (Nicolet iS50, Thermo Scientific, West Palm Beach, FL, USA).

### 2.5. XRD Measurement

XRD measurement of cellulose from pomelo fruitlets was performed by a Panalytical X’Pert PRO X-ray diffractometer (PANalytical B.V., Almelo, The Netherlands). The operating voltage was 40 kV, current was 40 mA, scanning rate was 2°/min, and angular range 2θ was from 5 to 40°. Crystallinity index CrI was calculated according to Equation (3) reported by Segal et al. [21].

(3) $$CrI\;(\%)=\frac{I_{002}-I_{am}}{I_{002}}\times 100\%,$$

where *I*_002_ is peak intensity at 2θ 22.6° corresponding to crystalline fraction; and *I_am_* is peak intensity at 2θ 18° corresponding to amorphous fraction.

### 2.6. SEM Observation

Surface morphology of cellulose from pomelo fruitlets was observed using a scanning electron microscopy (Hitachi S-4800, Hitachi Ltd., Tokyo, Japan). Cellulose from pomelo fruitlets were frozen, sublimated, gilded with a current of 40 mA, and then observed under accelerating voltage of 3.0 kV.

### 2.7. Particle Size Distribution

Particle size distribution of cellulose from pomelo fruitlets was analyzed by using a MasterSizer 2000 (Malvern Instruments Ltd., Malvern, UK). Anhydrous ethanol was adopted as the dispersant, stirring speed was 800 r/min, and determining temperature was 25 °C. The values of D_[4,3]_, D_[3,2]_, *d*_(10)_, *d*_(50)_, *d*_(90)_, and the specific surface area were recorded, where D_[4,3]_ is the volume-weighted diameter, D_[3,2]_ is the surface-weighted diameter, and *d*_(10)_, *d*_(50)_, and *d*_(90)_ represent the average diameter at which 10%, 50%, and 90% of the particles are smaller, respectively [12,22].

### 2.8. Water- and Oil-Holding Capacities Analysis

Water-holding capacity (WHC) and oil-holding capacity (OHC) of cellulose from pomelo fruitlets were determined using a previous method with minor modifications [23]. In brief, 1.00 g of cellulose from pomelo fruitlets was mixed with 25 mL distilled water or soybean oil for 1 h, and then centrifuged at 3000× *g* for 15 min. The supernatant was removed and the residue was weighed. Calculation of WHC and OHC was based on Equations (4) and (5),

(4) $$\mathrm{WHC}\;(g/g)=\frac{W_{w}}{W_{T}},$$

(5) $$\mathrm{OHC}\;(g/g)=\frac{W_{o}}{W_{T}},$$

where *W_w_* is the residue weight of cellulose/water solution after centrifugation, g; *W_o_* is the residue weight of cellulose/oil solution after centrifugation, g; and *W_T_* is the weight of the used cellulose, g.

### 2.9. Antioxidant Activities In Vitro

Antioxidant activities in vitro of cellulose from pomelo fruitlets were evaluated by 1,1-Diphenyl-2-picrylhydrazyl (DPPH) radical scavenging activity and ABTS radical scavenging activity according to the method reported previously [20].

### 2.10. Statistical Analysis

All experiments were conducted three times in parallel, and results were expressed as mean ± standard deviations. The data were evaluated by one-way analysis of variance using the SPSS 18.0 system. Statistical significance was set at *p* < 0.05.

## 3. Results

### 3.1. Yield and Purity of Cellulose from Pomelo Fruitlets

Cellulose CAA, CA, and CUA were prepared from pomelo fruitlets by acidic-alkaline hydrogen peroxide hydrolysis, alkaline hydrogen peroxide hydrolysis, and ultrasonic assisted alkaline hydrogen peroxide hydrolysis, respectively. Table 1 shows the yield and purity of cellulose CAA, CA, and CUA. Through acidic hydrolysis combined with alkaline hydrogen peroxide hydrolysis, the yield of cellulose CAA (8.64 ± 1.34%) and its purity (65.42 ± 3.55%) was the lowest among three samples (*p* < 0.05). The yield of cellulose CUA was close to that of cellulose CA, approximately 15%, which increased by 82.75% as compared to CA (*p* ≥ 0.05). The purity of cellulose CUA was up to 82.71 ± 3.37%, which was 26.42% higher than that of CA (*p* < 0.05). Furthermore, comparing extraction times of these three methods for producing cellulose, ultrasonic assisted alkaline hydrogen peroxide hydrolysis took less time (40 min) than acidic-alkaline hydrogen peroxide hydrolysis (270 min) or alkaline hydrogen peroxide hydrolysis (240 min). The results suggest that ultrasonic assisted alkaline hydrogen peroxide hydrolysis is a promising and efficient method to prepare cellulose from pomelo fruitlets with high yield and purity.

Many previous studies have reported that ultrasound-assisted pretreatment combining with chemicals such as alkali and dilute acid can effectively deconstruct cellulose and lignocellulosic biomasses [24,25,26]. In the reaction medium, ultrasound causes the generation of a pressure wave, including solvent molecules expanding, imploding, and releasing enormous amount of energy. This leads to the generation of active free radicals that help in delignification [24,25,26]. Therefore, such a delignification effect of ultrasound was the main reason for the highest purity of cellulose from pomelo fruitlets by ultrasonic assisted alkaline hydrogen peroxide hydrolysis in our study. During acidic-alkaline hydrogen peroxide hydrolysis, the treating time of NaOH and H_2_O_2_ to remove non-cellulose components such as hemicellulose was shorter than that of alkaline hydrogen peroxide hydrolysis. This might be attributed to its lower purity of cellulose CAA. A previous study suggested that acid hydrolysis can make cellulose molecules shorter and more accessible for chemicals or complete hydrolysis to produce glucose [27]. Such over-hydrolysis of cellulose induced by excessive acid treatment might be related to the lower yield of cellulose CAA from pomelo fruitlets in our study. Overall, the results implied that the delignification effect of ultrasound can improve yield and purity of cellulose from pomelo fruitlets with shorter extraction time, whereas unmanageable excessive acid treatment tends to reduce the yield of cellulose and impede the removal of impurities.

### 3.2. FT-IR Analysis of Cellulose from Pomelo Fruitlets

**Figure 1:**
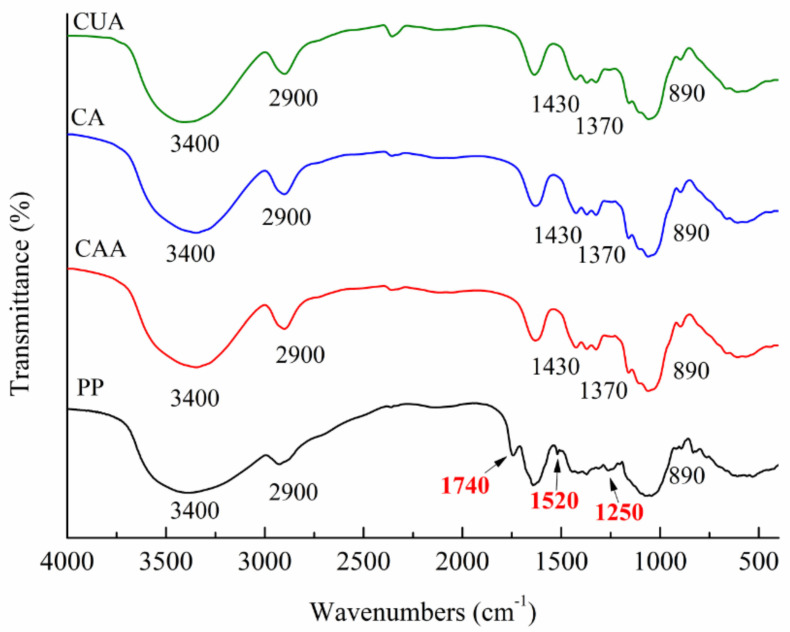
FT-IR spectra of cellulose from pomelo fruitlets. CAA, CA, and CUA are cellulose prepared by acidic-alkaline hydrogen peroxide hydrolysis, alkaline hydrogen peroxide hydrolysis, and ultrasonic assisted alkaline hydrogen peroxide hydrolysis, respectively. PP is native pomelo fruitlet powder.

Figure 1 shows FT-IR spectra of three types of cellulose from pomelo fruitlets using different extraction methods. Native pomelo fruitlet powder was used as a control. It exhibited main peaks at around 3400, 2900, 1740, 1520, 1250, and 890 cm^−1^. According to previous studies, peak at about 1740 cm^−1^ is caused by the carbonyl (C=O) unconjugated stretching vibration of ester and carbonyl group of hemicellulose present in pomelo fruit fibers [18]. This peak also belongs to the fingerprint region of pectin [28]. In our studies, the peak at approximately 1740 cm^−1^ disappeared in FT-IR spectra of cellulose CAA, CA, and CUA, suggesting the removal of most hemicellulose and pectin. The characteristic peak at 1520–1510 cm^−1^ is attributed to the aromatic –C=C– stretch of the aromatic rings of lignin [29]. The characteristic peak at 1300–1200 cm^−1^ is induced by C–O out-of-plane stretching of the aryl group of lignin and hemicellulose [18]. The peaks at 1520 and 1250 cm^−1^ of three cellulose could not be observed as compared to native pomelo fruitlet powders. This indicated that most of the lignin in pomelo fruitlet had been successfully removed for these three cellulose samples. The above results demonstrated the successful preparation of cellulose from pomelo fruitlets.

In addition, cellulose CAA, CA, and CUA showed similar absorption peaks associated with the characteristics of cellulose (type I), around 3400, 2900, 1430, 1370, and 890 cm^−1^. A large absorption peak at 3300–3500 cm^−1^ is related to O–H groups, whereas the peak at 2900 cm^−1^ is assigned as the C–H stretching vibrations. Peaks at 1430 and 1370 cm^−1^ are assigned as the C–O–H bending. The peak at 890 cm^−1^ are assigned as C–O–C stretching vibrations of the characteristic β-(1→4)-glycosidic linkage [30]. The similar peaks among cellulose CAA, CA, and CUA suggested that different extraction methods do not significantly affect the chemical structure of cellulose from pomelo fruitlets.

### 3.3. XRD Analysis of Cellulose from Pomelo Fruitlets

**Figure 2:**
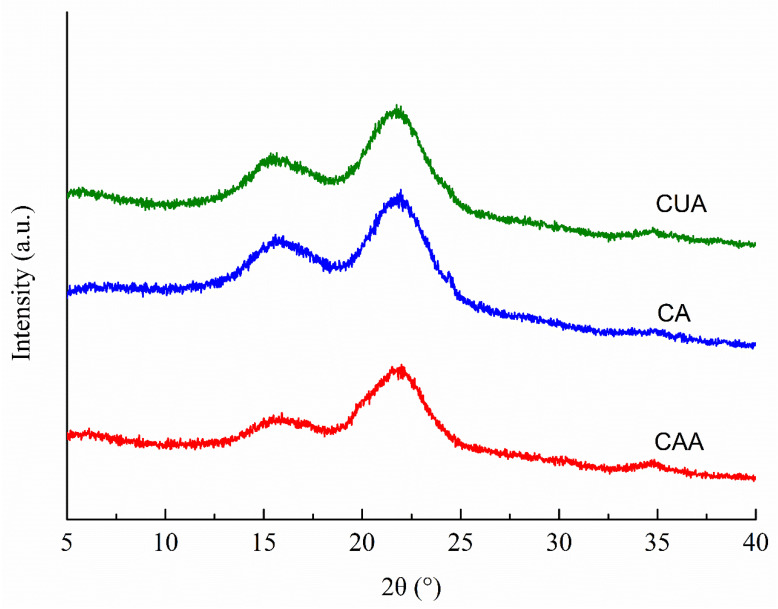
XRD patterns of cellulose from pomelo fruitlets. CAA, CA, and CUA are cellulose prepared by acidic-alkaline hydrogen peroxide hydrolysis, alkaline hydrogen peroxide hydrolysis, and ultrasonic assisted alkaline hydrogen peroxide hydrolysis, respectively.

Figure 2 shows the XRD patterns of three cellulose from pomelo fruitlets. All cellulose showed the typical cellulose-Ⅰ polymorphic structure broad bands at 2θ about 16.5°, 22.6°, and 34.2°, which were assigned to the crystalline planes with Miller indices of (110), (200), and (400), as suggested previously [19,31,32]. Hydrogen bonds between the cellulose chains and van der Waals forces between the glucose molecules result in the formation of crystalline regions in cellulose [33]. Among the diffraction peaks, one at around 22.6° is the main (200) cellulose I crystal peak. In our study, broad band at around 22.6° was sharper and narrower in cellulose CUA than in CA and CAA. It was suggested that ultrasonic assisted alkaline hydrogen peroxide hydrolysis promotes the formation of highly ordered cellulose crystallites in cellulose from pomelo fruitlets, probably through improving intra- and intermolecular hydrogen bonding. Accordingly, the estimated crystallinity index CrI of cellulose CUA was the highest with 44.26%, followed by CA with 31.7% and CAA with 30.2%. Qu et al. [34] also reported that ultrasonic pretreatment can promote crystallinity of cellulose fibers from wheat straw. This increased crystallinity is attributed to the removal of lignin and hemicellulose (the non-crystalline region, i.e., amorphous region). In our study, the highest purity of cellulose CUA (Table 1) also implied the better removal of impurities such as lignin and hemicellulose. This might be another reason for higher crystallinity in cellulose with ultrasonic assisted alkaline hydrogen peroxide hydrolysis. Moreover, the higher crystallinity in cellulose suggested that such materials are more suitable for extracting microcrystalline and nanocrystalline cellulose with high yield and are widely applied in various fields like pharmaceuticals, food processing, packaging, aerospace, and construction [35]. The findings suggested that ultrasonic assisted alkaline hydrogen peroxide hydrolysis is better for producing cellulose with higher crystallinity through removing lignin and hemicellulose in pomelo fruitlets, providing a useful method for improving the high-value application of pomelo fruitlets.

### 3.4. SEM Micrographs of Cellulose from Pomelo Fruitlets

**Figure 3:**
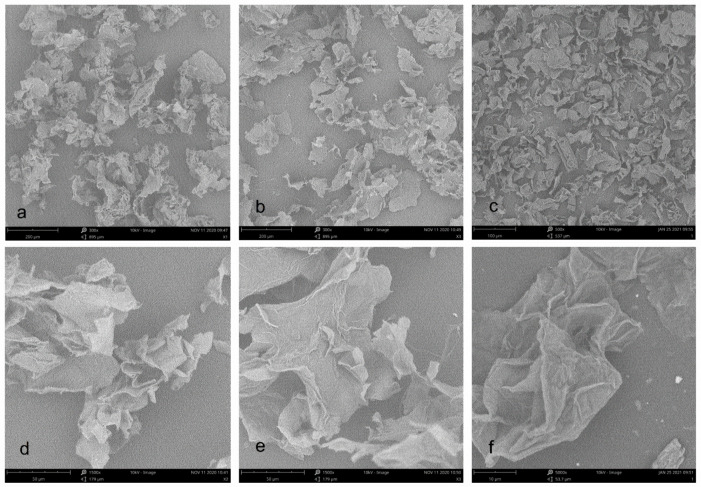
SEM images of cellulose from pomelo fruitlets: (**a**) CAA at 300× magnification; (**b**) CA at 300× magnification; (**c**) CUA at 500× magnification; (**d**) CAA at 1500× magnification; (**e**) CA at 1500× magnification; (**f**) CUA at 5000× magnification. CAA, CA, and CUA are cellulose prepared by acidic-alkaline hydrogen peroxide hydrolysis, alkaline hydrogen peroxide hydrolysis, and ultrasonic assisted alkaline hydrogen peroxide hydrolysis, respectively.

Figure 3 shows SEM micrographs of three cellulose from pomelo fruitlets. At 300× magnification, the morphology of cellulose CAA and CA were very similar to each other. They all have a foliated structure with the appearance of irregular curls. Compared with CA and CAA, the size of cellulose CUA was obviously reduced even at 500× magnification. At 1500× magnification, cellulose CAA displayed more irregular curls than CA, whereas cellulose CUA was too small to be clearly observed at this magnification. Thus, observation of cellulose CUA was performed at 5000× magnification. Cellulose CUA had a more distinct folded structure with a rougher surface than cellulose CA and CAA. Similar to a previous report, the surface of ultrasound treated cellulose sample is rather rough and appears ruptured with the formation of pores, resulting from simultaneous disintegration and rupturing of surfaces caused by NaOH and ultrasound, especially the effect of the implosion of microbubbles during sonication [25]. In summary, the results showed that ultrasonic assisted alkaline hydrogen peroxide hydrolysis facilitate the formation of microfiber in cellulose from pomelo fruitlet, with reduction of particle size.

### 3.5. Particle Size Analysis of Cellulose from Pomelo Fruitlets

**Figure 4:**
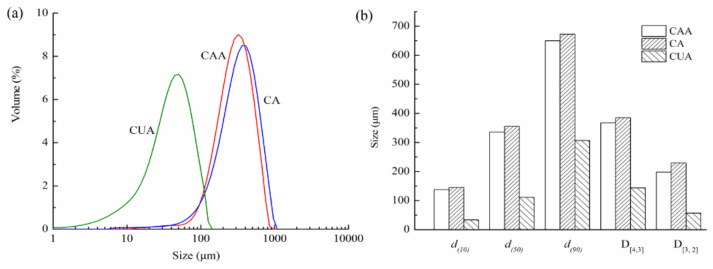
Particle size distribution (**a**) and size parameters (**b**) of cellulose from pomelo fruitlets.

Figure 4 shows particle size distribution of cellulose from pomelo fruitlets. Particle size distribution curves of three cellulose were all single and narrow peak, indicating that particle size of cellulose had uniformly distributed granular diameters. The volume-weighted diameter D_[4,3]_ of cellulose CUA was 143.92 μm, much smaller than cellulose CA (367.56 μm) and CAA (385.02 μm) (*p* < 0.05). Additionally, particle sizes of 50% CAA, CA, and CUA (i.e., *d*_(50)_) were less than 336.14, 355.46, and 110.98 μm, respectively. The differences of D_[3,2]_, *d*_(10)_, and *d*_(90)_ among these cellulose were similar to D_[4,3]_ and *d*_(50)_. These findings were consistent with the SEM observations, confirming that ultrasonic treatment can effectively reduce the particle size of cellulose from pomelo fruitlets. Zianor et al. [36] used ultrasound-assisted acid hydrolysis to separate cellulose from oil palm empty fruit bunch pulp and found that the diameter of cellulose is reduced from about 100 nm to 30–40 nm after ultrasonic treatment. The authors claimed that ultrasound effectively promotes the infiltration of acid molecules into cellulose, thus obtaining smaller cellulose particles. This is consistent with our results; it is reasonable to assume that ultrasonication promotes the action of alkaline hydrogen peroxide on reducing particle size of cellulose from pomelo fruitlets, contributing to the high yield and efficiency of the hydrolysis.

CAA, CA, and CUA are cellulose prepared by acidic-alkaline hydrogen peroxide hydrolysis, alkaline hydrogen peroxide hydrolysis, and ultrasonic assisted alkaline hydrogen peroxide hydrolysis, respectively.

### 3.6. WHC and OHC of Cellulose from Pomelo Fruitlets

Water-holding capacity (WHC) and oil-holding capacity (OHC) are important indicators for determining the functionality of cellulose. In general, cellulose with high WHC can promote human defecation, thus to reduce intestinal pressure and discharge intestinal toxins quickly [37]. Cellulose with high OHC is beneficial for reducing lipid absorption in digestion or preventing lipid loss during food processing [38]. Table 2 shows WHC and OHC of cellulose from pomelo fruitlets using different extraction methods. WHC of cellulose CUA (9.37 ± 0.10 g/g) was the highest among the three samples, followed by cellulose CA (8.29 ± 0.06 g/g) and then cellulose CAA (7.57 ± 0.05 g/g) (*p* < 0.05). The results showed that WHC of cellulose CUA was 23.77% and 13.02% higher than that of CAA and CA, respectively. Similarly, OHC of CAA, CA, and CUA were 4.42 ± 0.10, 4.72 ± 0.10, and 5.23 ± 0.10 g/g, respectively. Compared with CAA and CA, OHC of CUA increased by 18.32% and 10.80%, respectively. The results suggest that cellulose extracted from pomelo fruitlets by using ultrasonic assisted alkaline hydrogen peroxide hydrolysis has best performance of both WHC and OHC.

As reported previously, the structure, material type, and particle size of the product can greatly affect WHC and OHC of cellulose, where smaller particle size cause more hydrophilic and lipophilic groups exposure, leading to the increased contact area with water or oil [39,40,41,42]. Therefore, in our study, the highest WHC and OHC of cellulose CUA were closely related to its greater reduction of particle size caused by using ultrasonic assisted alkaline hydrogen peroxide hydrolysis as compared to acidic-alkaline hydrogen peroxide hydrolysis or alkaline hydrogen peroxide hydrolysis (Figure 3 and Figure 4). Scurria et al. (2021) [42] also reported the isolation of cellulose with good WHC from industrial citrus processing waste, where WHC of lemon cellulose was 8 g_water_/g_cell_ and that of grapefruit cellulose was 5 g_water_/g_cell_. Pomelo fruitlets are also citrus. In our study, besides the highest yield and purity, WHC of cellulose CUA from pomelo fruitlets was up to 9.37 ± 0.10 g/g, suggested high potential for developing cellulose with high WHC from pomelo fruitlets when appropriate extraction methods were applied. Therefore, ultrasonic assisted alkaline hydrogen peroxide hydrolysis was useful for producing functional cellulose from pomelo fruitlets, resulting in high, efficient utilization of this abundant biowaste.

### 3.7. Antioxidant Activities In Vitro of Cellulose from Pomelo Fruitlets

**Figure 5:**
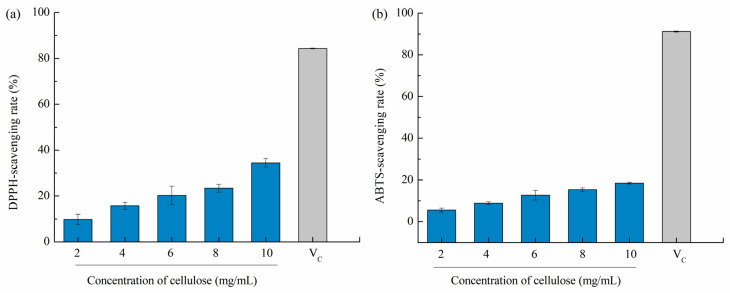
DPPH-scavenging rate (**a**) and ABTS-scavenging rate (**b**) of cellulose CUA from pomelo fruitlets. CUA is cellulose prepared by ultrasonic assisted alkaline hydrogen peroxide hydrolysis.

Overproduction of reactive oxygen species (such as hydroxyl radicals, superoxide radicals, peroxyl radicals, and NO radicals) inside cells tend to break the balance between oxidant generation and antioxidant systems, causing oxidative stress in the body and even inducing neurodegeneration diseases, cancer, atherosclerosis, and rheumatoid arthritis [43]. Therefore, natural free radical scavengers, e.g., polysaccharides, have gained considerable attention. Bian et al. (2013) [44] stated that xylooligosaccharides extracted from sugarcane bagasse hemicellulose has a dose-dependent antioxidant activity based on the DPPH method, and the rate of antioxidant activity increased to 84.5% with gradually increasing the concentration to 0.1–3 mg/mL. As reported by Liu et al. [15], DPPH-scavenging rates of 10 mg/mL dietary fiber were 12.09 ± 1.05% as compared to vitamin C, i.e., Vc (16.82 ± 0.82%), suggesting relatively superior antioxidant capacity. Therefore, we assumed that cellulose from pomelo fruitlets may also has potential antioxidant activities. In our study, cellulose CUA showed the highest yield, highest purity, and smallest particle size among different cellulose samples from pomelo fruitlets, thus it was used to evaluate antioxidant activities. As shown in Figure 5, the DPPH and ABTS scavenging capacities were analyzed in vitro. The DPPH-scavenging rate of cellulose CUA from pomelo fruitlets increased from 9.81 ± 2.22% to 34.47 ± 1.84% with increased cellulose concentration from 2 mg/mL to 10 mg/mL (*p* < 0.05). The ABTS-scavenging capacity of cellulose CUA from pomelo fruitlets was weaker than its DPPH-scavenging capacity, which was in the range of 5.53 ± 0.93% to 18.49 ± 0.44%. However, the DPPH and ABTS scavenging rate of cellulose CUA was much less than the control Vc group (84% and 91% at a concentration of 0.01 mg/mL). The results suggest cellulose from pomelo fruitlets extracted by ultrasonic assisted alkaline hydrogen peroxide hydrolysis does not exhibit superior antioxidant activities compared to Vc. The low antioxidant activities of cellulose from pomelo fruitlets may be caused by the oxidation of cellulose in the presence of H_2_O_2_ during extraction, but whether the oxidation of cellulose reduced their oxidation activity or whether cellulose itself has low oxidation activity remains to be further studied. To develop and evaluate cellulose from pomelo fruitlets extracted by ultrasonic assisted alkaline hydrogen peroxide hydrolysis as a multivalent functional food, the other bioactive potentials, e.g., hypoglycemic effect, should be studied in the future.

### 3.8. Future Prospects

Ultrasonic assisted alkaline hydrogen peroxide hydrolysis is a promising and efficient method to prepare high-purity cellulose from pomelo fruitlets (CUA). Higher WHC and OHC of cellulose CUA suggested it has a better effect on improving gut health though promoting human defecation and reducing lipid absorption. as discussed above. Thus, cellulose CUA from pomelo fruitlets can be used as a dietary fiber supplement in foods or a bioactive compound in drugs or health products, whereas the function and activity of CUA required comprehensive study. Moreover, due to the higher crystallinity and smaller particle size, cellulose CUA is suitable for preparation of microcrystalline and nanocrystalline cellulose products, which have already exerted a dramatic impact in biomedical, pharmaceutical, and food processing fields because of their unique rigidity, biodegradability, and biocompatibility. The physicochemical modification of inner crystalline layers is the key factor for those products’ colloidal properties (e.g., dispersion stability). Innovative means, e.g., directed assembly, tend to influence more advanced applications of cellulose from pomelo fruitlets.

## 4. Conclusions

The experimental observations revealed that pomelo fruitlets can be used to generate cellulose. Cellulose CUA extracted by ultrasonic assisted alkaline hydrogen peroxide hydrolysis had higher yield and purity with higher crystallinity and smaller particle size than those extracted by acidic-alkaline hydrogen peroxide hydrolysis or alkaline hydrogen peroxide hydrolysis. In addition, cellulose CUA exhibited superior water- and oil-holding capacities, which were related to its smaller particle size with increased surface area. Our findings suggest that ultrasonic assisted alkaline hydrogen peroxide hydrolysis is a promising and efficient method to prepare high-purity cellulose from pomelo fruitlets that are expected to be a food stabilizer and pharmaceutical additive. However, cellulose from pomelo fruitlets does not exhibit superior antioxidant activities as compared to Vc. To develop and evaluate cellulose from pomelo fruitlets as a multivalent functional food, other bioactive potentials, e.g., hypoglycemic effect, should be studied in the future. The result can provide knowledge for efficient production of cellulose from pomelo fruitlets using appropriate extraction methods and improve the utilization of pomelo fruitlets.

## Figures and Tables

**Table 1:** Yield and purity of cellulose from pomelo fruitlets.

Treatment	Yield (%)	Purity (%)
CAA	8.64 ± 1.34 ^b^	65.42 ± 3.55 ^c^
CA	15.23 ± 1.14 ^a^	75.52 ± 4.73 ^b^
CUA	15.79 ± 1.18 ^a^	82.71 ± 3.37 ^a^

^1^ CAA, CA and CUA are cellulose prepared by acidic-alkaline hydrogen peroxide hydrolysis, alkaline hydrogen peroxide hydrolysis, and ultrasonic assisted alkaline hydrogen peroxide hydrolysis, respectively. ^2^ Different superscript lowercase letters (a, b, c) among CAA, CA, and CUA indicate statistical significance between different samples at *p* < 0.05.

**Table 2:** Water- and oil-holding capacities of cellulose from pomelo fruitlets.

Treatment	Water-Holding Capacities (g/g)	Oil-Holding Capacities (g/g)
CAA	7.57 ± 0.05 ^c^	4.42 ± 0.10 ^c^
CA	8.29 ± 0.06 ^b^	4.72 ± 0.10 ^b^
CUA	9.37 ± 0.10 ^a^	5.23 ± 0.10 ^a^

^1^ CAA, CA, and CUA are cellulose prepared by acidic-alkaline hydrogen peroxide hydrolysis, alkaline hydrogen peroxide hydrolysis, and ultrasonic assisted alkaline hydrogen peroxide hydrolysis, respectively. ^2^ Different superscript lowercase letters (a, b, c) among CAA, CA, and CUA indicate statistical significance between different samples at *p* < 0.05.

## References

[1] He X., Lu W., Sun C., Khalesi H., Mata A., Andaleeb R., Fang Y. (2021). Cellulose and cellulose derivatives: Different colloidal states and food-related applications. Carbohydr. Polym..

[2] Baghel R.S., Reddy C.R.K., Singh R.P. (2021). Seaweed-based cellulose: Applications, and future perspectives. Carbohydr. Polym..

[3] Huang S., Liu X., Chang C., Wang Y. (2020). Recent developments and prospective food-related applications of cellulose nanocrystals: A review. Cellulose.

[4] Sabbagh F., Muhamad I.I. (2017). Acrylamide-based hydrogel drug delivery systems: Release of Acyclovir from MgO nanocomposite hydrogel. J. Taiwan Inst. Chem. E.

[5] Ait Benhamou A., Kassab Z., Nadifiyine M., Hamid Salim M., Sehaqui H., Moubarik A., El Achaby M. (2021). Extraction, characterization and chemical functionalization of phosphorylated cellulose derivatives from Giant Reed Plant. Cellulose.

[6] Owolabi A.F., Haafiz M.K.M., Hossain M.S., Hussin M.H., Fazita M.R.N. (2017). Influence of alkaline hydrogen peroxide pre-hydrolysis on the isolation of microcrystalline cellulose from oil palm fronds. Int. J. Biol. Macromol..

[7] Haafiz M.M., Eichhorn S.J., Hassan A., Jawaid M. (2013). Isolation and characterization of microcrystalline cellulose from oil palm biomass residue. Carbohydr. Polym..

[8] Maiti S., Jayaramudu J., Das K., Reddy S.M., Sadiku R., Ray S.S., Liu D. (2013). Preparation and characterization of nano-cellulose with new shape from different precursor. Carbohydr. Polym..

[9] Morais J.P.S., Rosa M.D.F., Filho M.D.S., Nascimento L.D., do Nascimento D.M., Cassales A.R. (2013). Extraction and characterization of nanocellulose structures from raw cotton linter. Carbohydr. Polym..

[10] Asem M., Jimat D.N., Jafri N.H.S., Nawawi W.M.F.W., Azmin N.F.M., Wahab M.F.A. (2021). Entangled cellulose nanofibers produced from sugarcane bagasse via alkaline treatment, mild acid hydrolysis assisted with ultrasonication. JKSUES.

[11] Sabbagh F., Muhamad I.I., Pa’e N., Hashim Z., Mondal M. (2019). Strategies in Improving Properties of Cellulose-Based Hydrogels for Smart Applications. Cellulose-Based Superabsorbent Hydrogels.

[12] Zheng M., Hong J., Li M., He H., Jiang Z., Ni H., Li Q. (2021). Effects of particle sizes on structural and physicochemical properties of pomelo peel powders. J. Food Process. Pres..

[13] Wei H., He C., Zhang S., Xiong H., Ni H., Li Q. (2021). Effects of four storage conditions on the sugar content, acidity, and flavor of “Guanxi” honey pomelo. J. Food Process. Preserv..

[14] Liu H., Fang Y., Zou C. (2021). Pomelo polysaccharide extract inhibits oxidative stress, inflammation, and mitochondrial apoptosis of *Epinephelus coioides*. Aquaculture.

[15] Liu H., Zeng X., Huang J., Yuan X., Wang Q., Ma L. (2021). Dietary fiber extracted from pomelo fruitlets promotes intestinal functions, both in vitro and *in vivo*. Carbohydr. Polym..

[16] Liu Y., Liu A., Ibrahim S.A., Yang H., Huang W. (2018). Isolation and characterization of microcrystalline cellulose from pomelo peel. Int. J. Biol. Macromol..

[17] Tang F., Yu H., Yassin Hussain Abdalkarim S., Sun J., Fan X., Li Y., Zhou Y., Chiu Tam K. (2020). Green acid-free hydrolysis of wasted pomelo peel to produce carboxylated cellulose nanofibers with super absorption/flocculation ability for environmental remediation materials. Chem. Eng. J..

[18] Yongvanich N. (2015). Isolation of Nanocellulose from Pomelo Fruit Fibers by Chemical Treatments. J. Nat. Fibers.

[19] Chowdhury Z.Z., Abd Hamid S.B. (2016). Preparation and Characterization of Nanocrystalline Cellulose using Ultrasonication Combined with a Microwave-assisted Pretreatment Process. BioResources.

[20] Zheng M., Liu X., Chuai P., Jiang Z., Zhu Y., Zhang B., Ni H., Li Q. (2021). Effects of crude fucoidan on physicochemical properties, antioxidation and bacteriostasis of surimi products. Food Control.

[21] Segal L., Creely J., Martin A., Conrad C. (1959). An Empirical Method for Estimating the Degree of Crystallinity of Native Cellulose Using the X-Ray Diffractometer. Text. Res. J..

[22] Zheng M., Ye A., Singh H., Zhang Y. (2021). The in vitro digestion of differently structured starch gels with different amylose contents. Food Hydrocolloid..

[23] Li B., Yang W., Nie Y., Kang F., Goff H.D., Cui S.W. (2019). Effect of steam explosion on dietary fiber, polysaccharide, protein and physicochemical properties of okara. Food Hydrocolloid..

[24] SriBala G., Chennuru R., Mahapatra S., Vinu R. (2016). Effect of alkaline ultrasonic pretreatment on crystalline morphology and enzymatic hydrolysis of cellulose. Cellulose.

[25] Luo J., Fang Z., Smith R.L. (2014). Ultrasound-enhanced conversion of biomass to biofuels. Prog. Energ. Combust..

[26] Sasmal S., Goud V., Mohanty K. (2012). Ultrasound assisted lime pretreatment of lignocellulosic biomass toward bioethanol production. Energ. Fuels.

[27] Trygg J., Fardim P. (2011). Enhancement of cellulose dissolution in water-based solvent via ethanol-hydrochloric acid pretreatment. Cellulose.

[28] Ilangovan M., Guna V., Prajwal B., Jiang Q., Reddy N. (2020). Extraction and characterisation of natural cellulose fibers from *Kigelia africana*. Carbohydr. Polym..

[29] Elanthikkal S., Gopalakrishnapanicker U., Varghese S., Guthrie J.T. (2010). Cellulose microfibres produced from banana plant wastes: Isolation and characterization. Carbohydr. Polym..

[30] Ibrahim M.M., El-Zawawy W.K., Jüttke Y., Koschella A., Heinze T. (2013). Cellulose and microcrystalline cellulose from rice straw and banana plant waste: Preparation and characterization. Cellulose.

[31] Sankhla S., Sardar H.H., Neogi S. (2021). Greener extraction of highly crystalline and thermally stable cellulose micro-fibers from sugarcane bagasse for cellulose nano-fibrils preparation. Carbohydr. Polym..

[32] Fortunati E., Benincasa P., Balestra G.M., Luzi F., Mazzaglia A., Del Buono D., Puglia D., Torre L. (2016). Revalorization of barley straw and husk as precursors for cellulose nanocrystals extraction and their effect on PVA CH nanocomposites. Ind. Crop. Prod..

[33] Bansal P., Hall M., Realff M.J., Lee J.H., Bommarius A.S. (2010). Multivariate statistical analysis of X-ray data from cellulose: A new method to determine degree of crystallinity and predict hydrolysis rates. Bioresour. Technol..

[34] Qu R., Tang M., Wang Y., Li D., Wang L. (2021). TEMPO-oxidized cellulose fibers from wheat straw: Effect of ultrasonic pretreatment and concentration on structure and rheological properties of suspensions. Carbohydr. Polym..

[35] Hachaichi A., Kouini B., Kian L.K., Asim M., Jawaid M. (2021). Extraction and Characterization of Microcrystalline Cellulose from Date Palm Fibers using Successive Chemical Treatments. J. Polym. Environ..

[36] Zianor A., Beg M.D.H., Rosli M.Y., Ramli R., Junadi N. (2017). Spherical nanocrystalline cellulose (NCC) from oil palm empty fruit bunch pulp via ultrasound assisted hydrolysis. Carbohydr. Polym..

[37] Eswaran S., Muir J., Chey W.D. (2013). Fiber and Functional Gastrointestinal Disorders. Am. J. Gastroenterol..

[38] Chen Y., Lin Y.-J., Nagy T., Kong F., Guo T.L. (2020). Subchronic exposure to cellulose nanofibrils induces nutritional risk by non-specifically reducing the intestinal absorption. Carbohydr. Polym..

[39] Rizzello C.G., Coda R., Mazzacane F., Minervini D., Gobbetti M. (2012). Micronized by-products from debranned durum wheat and sourdough fermentation enhanced the nutritional, textural and sensory features of bread. Food Res. Int..

[40] Chen J., Gao D., Yang L., Gao Y. (2013). Effect of microfluidization process on the functional properties of insoluble dietary fiber. Food Res. Int..

[41] He H., An F., Wang Y., Wu W., Huang Z., Song H. (2021). Effects of pretreatment, NaOH concentration, and extraction temperature on the cellulose from *Lophatherum Gracile Brongn*. Int. J. Biol. Macromol..

[42] Scurria A., Albanese L., Pagliaro M., Zabini F., Giordano F., Meneguzzo F., Ciriminna R. (2021). CytroCell: Valued Cellulose from Citrus Processing Waste. Molecules.

[43] Sanjeewa K.K.A., Kang N., Ahn G., Jee Y., Kim Y.T., Jeon Y.J. (2018). Bioactive potentials of sulfated polysaccharides isolated from brown seaweed *Sargassum* spp in related to human health applications: A review. Food Hydrocolloid..

[44] Bian J., Peng F., Peng X., Peng P., Xu F., Sun R. (2013). Structural features and antioxidant activity of xylooligosaccharides enzymatically produced from sugarcane bagasse. Bioresour. Technol..

